# Newborn Screening Program for Mucopolysaccharidosis Type II and Long-Term Follow-Up of the Screen-Positive Subjects in Taiwan

**DOI:** 10.3390/jpm12071023

**Published:** 2022-06-21

**Authors:** Hsiang-Yu Lin, Ya-Hui Chang, Chung-Lin Lee, Yuan-Rong Tu, Yun-Ting Lo, Pei-Wen Hung, Dau-Ming Niu, Mei-Ying Liu, Hsin-Yun Liu, Hsiao-Jan Chen, Shu-Min Kao, Li-Yun Wang, Huey-Jane Ho, Chih-Kuang Chuang, Shuan-Pei Lin

**Affiliations:** 1 Department of Pediatrics, MacKay Memorial Hospital, Taipei 10449, Taiwan; lxc46199@ms37.hinet.net (H.-Y.L.); wish1001026@gmail.com (Y.-H.C.); clampcage@gmail.com (C.-L.L.); 2 Department of Medical Research, MacKay Memorial Hospital, Taipei 10449, Taiwan; likemaruko@hotmail.com; 3 The Rare Disease Center, MacKay Memorial Hospital, Taipei 10449, Taiwan; andy11tw.e347@mmh.org.tw (Y.-T.L.); hig1812@yahoo.com.tw (P.-W.H.); 4 Department of Medicine, MacKay Medical College, New Taipei City 25245, Taiwan; 5 MacKay Junior College of Medicine, Nursing and Management, Taipei 11260, Taiwan; 6 Department of Medical Research, China Medical University Hospital, China Medical University, Taichung 40402, Taiwan; 7 Department of Pediatrics, Taipei Veterans General Hospital, Taipei 11217, Taiwan; dmniu1111@yahoo.com.tw; 8 The Chinese Foundation of Health, Neonatal Screening Center, Taipei 10699, Taiwan; meiying@cfoh.tw (M.-Y.L.); hyliu@cfoh.tw (H.-Y.L.); hjchen@cfoh.tw (H.-J.C.); kao@cfoh.tw (S.-M.K.); 9 Taipei Institute of Pathology, Neonatal Screening Center, Taipei 10374, Taiwan; yurilarc@gmail.com (L.-Y.W.); juju5113@gmail.com (H.-J.H.); 10 College of Medicine, Fu-Jen Catholic University, Taipei 24205, Taiwan; 11 Department of Infant and Child Care, National Taipei University of Nursing and Health Sciences, Taipei 11219, Taiwan

**Keywords:** enzyme replacement therapy, genotype-phenotype correlation, glycosaminoglycans, hematopoietic stem cell transplantation, mucopolysaccharidosis II (MPS II), newborn screening program

## Abstract

Background: Mucopolysaccharidosis II (MPS II) is an X-linked disorder resulting from a deficiency in lysosomal enzyme iduronate-2-sulfatase (IDS), which causes the accumulation of glycosaminoglycans (GAGs) in the lysosomes of many tissues and organs, leading to progressive cellular dysfunction. An MPS II newborn screening program has been available in Taiwan since 2015. The aim of the current study was to collect and analyze the long-term follow-up data of the screen-positive subjects in this program. Methods: From August 2015 to April 2022, 548,624 newborns were screened for MPS II by dried blood spots using tandem mass spectrometry, of which 202 suspected infants were referred to our hospital for confirmation. The diagnosis of MPS II was confirmed by IDS enzyme activity assay in leukocytes, quantitative determination of urinary GAGs by mass spectrometry, and identification of the *IDS* gene variant. Results: Among the 202 referred infants, 10 (5%) with seven *IDS* gene variants were diagnosed with confirmed MPS II (Group 1), 151 (75%) with nine *IDS* gene variants were classified as having suspected MPS II or pseudodeficiency (Group 2), and 41 (20%) with five *IDS* gene variants were classified as not having MPS II (Group 3). Long-term follow-up every 6 months was arranged for the infants in Group 1 and Group 2. Intravenous enzyme replacement therapy (ERT) was started in four patients at 1, 0.5, 0.4, and 0.5 years of age, respectively. Three patients also received hematopoietic stem cell transplantation (HSCT) at 1.5, 0.9, and 0.6 years of age, respectively. After ERT and/or HSCT, IDS enzyme activity and the quantity of urinary GAGs significantly improved in all of these patients compared with the baseline data. Conclusions: Because of the progressive nature of MPS II, early diagnosis via a newborn screening program and timely initiation of ERT and/or HSCT before the occurrence of irreversible organ damage may lead to better clinical outcomes. The findings of the current study could serve as baseline data for the analysis of the long-term effects of ERT and HSCT in these patients.

## 1. Introduction

Mucopolysaccharidosis type II (MPS II; Hunter syndrome; OMIM 309900) is an X-linked recessive lysosomal storage disorder caused by a deficiency of iduronate-2-sulfatase (IDS) activity, which is involved in the lysosomal degradation of the glycosaminoglycans (GAGs), dermatan sulfate (DS) and heparan sulfate (HS). The accumulation of GAGs in lysosomes can contribute to progressive cellular dysfunction in multiple tissues and organs [1,2,3,4]. The clinical manifestations of MPS may emerge in patients from early to late childhood or even in early adulthood depending on the severity of the MPS phenotype. Patients with MPS II are clinically classified into two forms, a severe form and a mild form [5,6]. The severe form usually manifests between 18 months and 4 years of age, with coarse facial features, short stature [7], developmental delay [8], adenotonsillar hypertrophy, recurrent ear, nose, and throat infections, hearing impairment, airway obstruction, pulmonary function impairment [9], cardiac valve dysplasia, cardiomyopathy, hepatosplenomegaly, inguinal and umbilical hernias, skeletal dysplasia (dysostosis multiplex), kyphoscoliosis, joint stiffness, hyperactivity, and profound cognitive impairment. Death from cardiac and respiratory failure or respiratory infections usually occurs before the second decade of life [5]. Patients with the mild form do not have cognitive impairment. Their somatic involvement can range from early onset and severe to later onset with much less severity, and less severely affected patients may survive to their fifth or sixth decade of life [1,6].

The predominance of MPS II among the various types of MPSs in Taiwan (52%) is similar to other Asian countries, including China (47.4%), Japan (55%), and South Korea (54.6%). However, in most Caucasian populations, MPS III or MPS I is the most dominant MPS type. The birth prevalence of MPS II differs among different populations, i.e., 1.07 per 100,000 live births in Taiwan; 0.74 per 100,000 live births in South Korea; 0.84 per 100,000 live births in Japan; and 0.64, 0.46, and 0.67 per 100,000 live births in Germany, Poland, and The Netherlands, respectively. A founder effect may explain the variations in MPS type prevalence across various ethnic populations [10,11].

Hematopoietic stem cell transplantation (HSCT) and enzyme replacement therapy (ERT) are the principal therapies for MPS II, which can alleviate or prevent the development of severe clinical symptoms. HSCT is currently the only therapeutic option to prevent progressive neurodegenerative disorders in MPS I, II, and VII. However, its application is restricted by the potential risk of graft failure and transplantation-related morbidity and mortality [12,13,14]. ERT is now available for patients with MPS II, and it has been verified to significantly reduce urinary GAG quantities and substantially improve joint mobility, physiological activities, endurance, and quality of life [15]. Additional treatments for MPS II, including substrate reduction therapy, chaperone therapy, and gene therapy, are presently in clinical trials [16].

The clinical symptoms of MPS II develop progressively, and as with other types of MPS disorders, many manifestations may be irreversible once apparent. Previous sibling studies have demonstrated that early treatment may lead to better clinical outcomes [17,18]. Except for cases with a family history of the disease, presymptomatic MPS II can only be detected by newborn screening. An early diagnosis of MPS II through such newborn screening programs can promote more timely and appropriate management plans for these patients, and allow for more detailed genetic counseling for the family members. Newborn screening programs for MPS II are currently conducted in Taiwan, Illinois, and Missouri [19,20,21]. The pilot newborn screening program for MPS II was launched in Taiwan in August 2015, and infants who fail to pass the recheck at recall are referred to MacKay Memorial Hospital for a comprehensive confirmatory diagnosis. The median age at a confirmative diagnosis of MPS II can be as early as 0.2 years in an asymptomatic patient, which is much earlier than the 3.5 years reported in Taiwan for a symptomatic patient [6,19,22,23,24].

Accurate information of the specific *IDS* mutations involved in MPS II may help to clarify the relationships between genotype and phenotype in individual patients and identify female carriers. Since only a handful of reports have reported long-term follow-up data of screen-positive subjects, the purpose of the current study was to collect and analyze data on the newborn screening program for MPS II in Taiwan and long-term follow-up data of the screen-positive subjects. This could help to better understand the natural progression of this disease, as well as the beneficial effects of an early diagnosis and interventions for these patients.

## 2. Materials and Methods

### 2.1. Study Population

**Figure 1:**
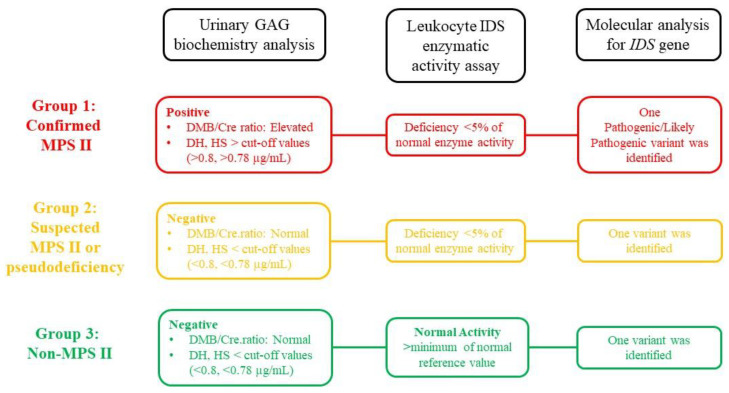
The referred infants from our MPS II newborn screening program were classified into three diagnostic groups as follows: Group 1: confirmed MPS II, Group 2: suspected MPS II or pseudodeficiency, and Group 3: non-MPS II according to the results of urinary GAG biochemistry tests, leukocyte IDS enzymatic activity assay, and molecular analysis of the *IDS* gene. MPS, mucopolysaccharidosis; GAG, glycosaminoglycan; IDS, iduronate-2-sulfatase; DS, dermatan sulfate; HS, heparan sulfate.

From August 2015 to April 2022, 548,624 newborns were screened for MPS II by detecting IDS enzyme activity in dried blood spots (DBSs) using a tandem-mass-spectrometry-based assay, of whom 202 suspected infants were referred to our hospital for confirmation. The diagnosis of MPS II was confirmed by IDS enzyme activity assay in leukocytes, two-dimensional electrophoresis of urinary GAGs, quantitative determination of DS, HS, keratan sulfate (KS), and chondroitin sulfate (CS) using liquid chromatography/tandem mass spectrometry (LC-MS/MS), and identification of *IDS* gene variants as previously described [19,25,26,27]. The definition of a true “positive” confirmatory diagnosis of MPS II was defined as a “positive” urinary biochemistry examination, particularly an elevated quantity of urinary GAG-derived disaccharides, deficient activity (less than 5% of normal activity) of leukocyte IDS enzyme, and identification of *IDS* gene variants by Sanger sequencing or next-generation sequencing. As described in our previous report [28], the IDS activity in extracts of COS-7 cells expressing novel mutant cDNA was also examined in the present study, i.e., for *IDS* gene variants c.659T > C, c.1513T > C, and c.805G > A. The infants were classified into three MPS diagnostic groups according to the following findings (Figure 1). Group 1 (confirmed MPS II) was defined as being “positive” for urinary GAG biochemistry examinations, “deficient” IDS enzymatic activity, and the identification of one hemizygous variant of the *IDS* gene. Group 2 (suspected MPS II or pseudodeficiency) was defined as “negative” for urinary GAG biochemistry examinations, “deficiency” of IDS enzymatic activity, and the identification of one hemizygous variant of the *IDS* gene. Group 3 (non-MPS II) was defined as “negative” for urinary GAG biochemistry examinations, “normal” for IDS enzymatic activity, and the identification of one hemizygous variant of the *IDS* gene. Physical examinations, bone X-ray, echocardiography, and abdominal ultrasonography were also performed for the referred infants to detect any emerging manifestations. For the infants in Group 1 and Group 2, long-term follow-up every 6 months was suggested and arranged. Because MPS II is an autosomal-recessive inherited disease, maternal-side senior male relative screening was also conducted. The current study was approved by the Institutional Review Board of MacKay Memorial Hospital (21CT013be), and written informed consent was obtained from all of the patients or their parents.

### 2.2. MS-Based Assay Used for the Screening on DBS

The MS-based assay is applied in Newborn Screening Centers for IDS enzyme activity in DBS. This method uses a substrate that is analogous in structure to the natural substrate and an isotope (deuterium) labeled internal standard designed to match the resulting expected product from the assay. The *m*/*z* (mass-to-charge ratio) of IDS substrate and IDS internal standard detected by tandem mass spectrometry were 767.23/643.31, and 648.34/643.31, respectively. The cut-off value was determined based on 30% of the mean activity, about 6.5 μmol/L/h for the first DBS, and 10% of the mean activity for the second DBS, about 2.2 μmol/L/h. The false-positivity rate of the screening assessment was lower than 0.50%. The criterion for determining a “suspected infant” in Newborn Screening Centers is defined as one specific enzyme activity in DBS filter paper being lower than the cut-off value in continuous blood sampling twice. Suspected infants are referred to MacKay Memorial Hospital for confirmation.

### 2.3. Leukocyte IDS Enzyme Activity by Fluorometric Assay

The protocols of IDS enzyme assays have been described previously [19,29]. Individual enzyme activity about 5% lower than normal was defined as a marked reduction in IDS enzyme activity, and this was the diagnostic basis to confirm MPS. The reference range for leukocyte IDS enzyme activity is 12.89–131.83 µmol/g protein/4 h.

### 2.4. Total GAG Quantification (Dimethylene Blue/Creatinine Ratio; DMB/Cre Ratio)

The method of calculating the DMB/Cre ratio has been described previously [24,30,31]. The normal reference values according to age group are: <6 months: <70.68 mg/mmol Cre; 6 months–2 years: <46.80 mg/mmol Cre; 2–17 years: <20.98 mg/mmol Cre; and >18 years: <12.62 mg/mmol Cre.

### 2.5. GAG-Derived Disaccharide Quantification by Tandem Mass Spectrometry Assay

Tandem mass spectrometry (liquid chromatography/tandem mass spectrometry; LC-MS/MS) has been widely used to quantify the levels of urinary GAG-derived disaccharides for MPS diagnosis. LC-MS/MS analysis was performed on an AB 4000 QTRAP LC-MS/MS System (AB Sciex, Foster City, CA, USA) equipped with a TurboIonSpray (electrospray ionization; ESI) and Agilent 1260 Infinity HPLC pump and autosampler (Agilent Technologies, Santa Clara, CA, USA). An Atlantis dC18 3 μm column (3.0 × 50 mm; Waters Corporation, Milford, MA, USA) was used for DS and HS analysis, and a Luna 5μm Silica column (50 × 2.0 mm; Phenomenex Inc., Torrance, CA, USA) was used for KS analysis. The principles of the LC-MS/MS assay for relevant GAG-derived disaccharides were performed using methanolysis (chemical hydrolysis) for CS, DS, and HS, and using keratanase II enzyme digestion for KS quantification. In brief, the GAGs were precipitated and then degraded to uronic acid-N-acetylhexosamine dimers. The *m*/*z* (mass to charge) of the parent ion and its daughter ion after collision was 426.1→236.2 for DS and 384.2→161.9 for HS. LC-MS/MS assay for KS-derived disaccharides has been performed by treatment of keratanase II (GlycoSyn; Lower Huf, New Zealand). N-acetylglucosamine linkages of the KS chain were cleaved and released Galβ1-4GlcNAc (N-acetylglucosamine) disaccharides with mono-sulfates. One particular disaccharide of KS was selected by monitoring the decay of the *m*/*z* 462 precursor to the *m*/*z* 97 production of Gal β1→4 GlcNAc(6 S) disaccharides derived from KS [19,26,27]. The cut-off values for DS, HS, and KS were made in which the cut-off values were <0.80 μg/mL for DS, <0.78 μg/mL for HS, and <7.90 μg/mL for KS. In this study, we used the CS-normalized method to calculate (μg/mL) instead of the creatinine-normalized method (μg/mg creatinine) that can effectively prevent false positives and false negatives for DS, HS, and KS quantification [27].

### 2.6. Nucleotide Variation Detected by Sanger Sequencing

For the molecular analysis, we performed Sanger sequencing instead of next-generation sequencing except when the variant was difficult to define or identify. The Sanger sequencing was performed based on the following protocols, including: (1). primer design and determination of melting temperature (T_m_) of the primers for PCR experiment; (2). PCR product was authorized for DNA sequencing analysis by a qualified biotechnology company (ISO/IEL 17025); (3). alignment of sequencing data; (4). mutation gene identification and determination; and (5). survey literatures and HGMD database for confirmation.

### 2.7. The IDS Activity in Extracts of COS-7 Cells Expressing IDS for Mutant cDNAs

Plasmids expressing IDS protein were made by cloning the *IDS* cDNA into a pCMV6-Entry-based Myc-DDK tagging vector (ORIGENE, RC219187). The novel mutations of the *IDS* gene were introduced into the wild-type *IDS* cDNA by site-directed mutagenesis. The COS-7 cells were transfected by pCMV6-Entry plasmids of wild-type *IDS* cDNA and the mutants using Lipofectamine 3000 (Invitrogen, Carlsbad, CA, USA) following the manufacturer’s protocol. After 24 h of incubation at 37 °C, the cells were harvested for IDS enzyme assays. These experiments were performed in triplicate [28].

### 2.8. Data and Statistical Analysis

The results of descriptive statistics are presented as the mean ± standard deviation or median value unless otherwise indicated.

## 3. Results

Among the 202 referred infants, 10 (5%) with seven *IDS* gene variants were diagnosed with confirmed MPS II (Group 1), 151 (75%) with nine *IDS* gene variants were classified as having suspected MPS II or pseudodeficiency (Group 2), and 41 (20%) with five *IDS* gene variants were classified as not having MPS II (Group 3) (Table 1).

**Figure 2:**
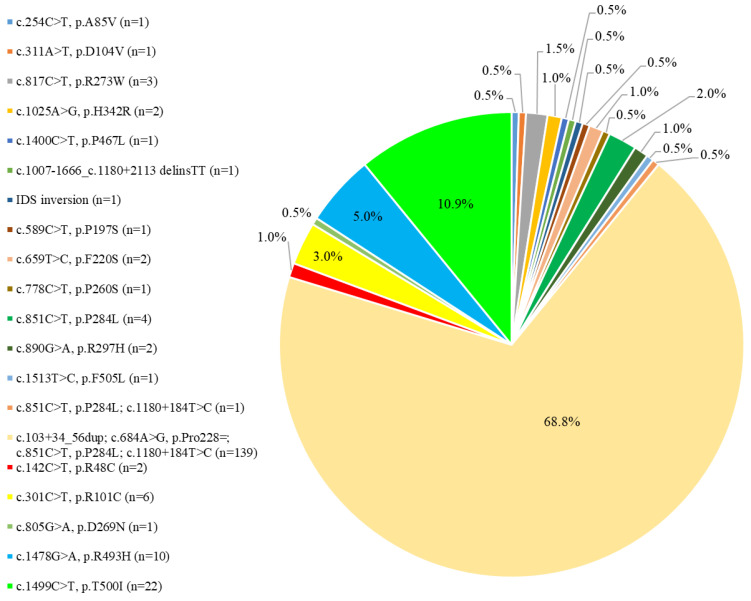
The numbers and percentages of the 21 *IDS* gene variants identified in the 202 infants in our MPS II newborn screening program.

**Figure 3:**
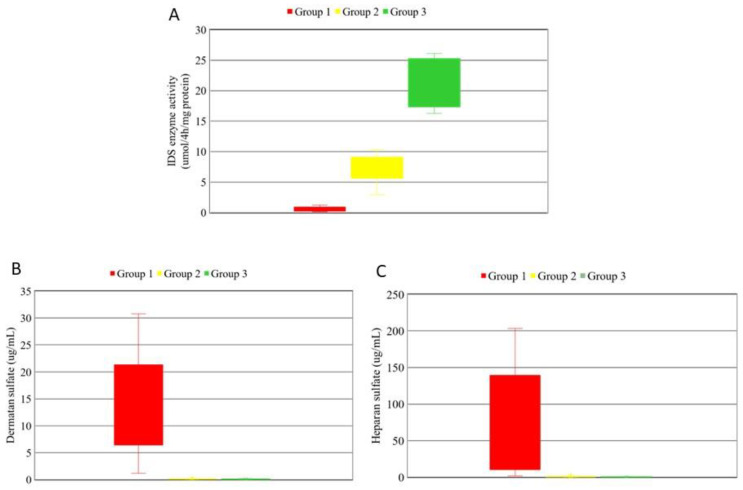
The IDS enzyme activity (**A**), urinary dermatan sulfate (**B**), and urinary heparan sulfate (**C**) quantities for the subjects of the three diagnostic groups. IDS, iduronate-2-sulfatase.

Figure 2 shows the numbers and percentages of the 21 *IDS* gene variants identified in the 202 infants in our MPS II newborn screening program. Among the 21 *IDS* gene variants, only five have been reported previously for clinically diagnosed MPS II, all of which were reported in the literature rather than from newborn screening programs for MPS II, namely c.1400C > T, p.P467L [32,33], c.1007–1666_c.1180 + 2113 delinsTT [34,35], IDS inversion [18,36], c.851C > T, p.P284L [37], and c.301C > T, p.R101C [38]. Figure 3 shows the IDS enzyme activity and urinary DS and HS quantities for the 202 infants in the three diagnostic groups. Long-term follow-up every 6 months was arranged for the infants in Group 1 and Group 2. 

### 3.1. Group 1: Confirmed MPS II

**Figure 4:**
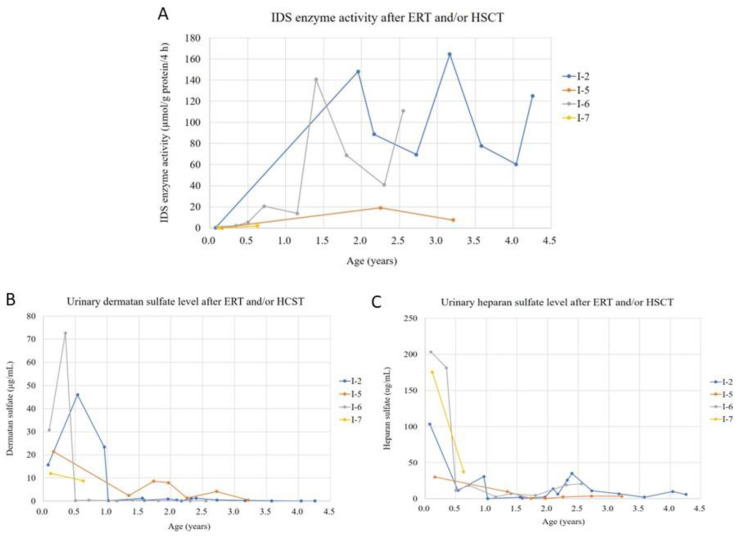
The IDS enzyme activity (**A**), urinary dermatan sulfate (**B**), and urinary heparan sulfate (**C**) quantities for four MPS II patients diagnosed through the newborn screening program who received early ERT and/or HSCT. After ERT and/or HSCT, all results significantly improved compared with the baseline data. Intravenous ERT was started in four patients (No. I-2, I-5, I-6, I-7) at 1, 0.5, 0.4, and 0.5 years of age, respectively. Patients No. I-2, I-6, and I-7 also received HSCT at 1.5, 0.9, and 0.6 years of age, respectively. IDS, iduronate-2-sulfatase. ERT, enzyme replacement therapy; HSCT, hematopoietic stem cell transplantation.

Seven *IDS* gene variants were identified in this group. Ten infants in this group were “positive” for urinary GAG biochemistry examinations, “deficient” for IDS enzymatic activity, and had at least one hemizygous variant of the *IDS* gene. ERT and/or HSCT were provided as early as possible, and long-term follow-up every 6 months was arranged for these infants. Intravenous ERT was started in four patients (No. I-2, I-5, I-6, I-7) at 1, 0.5, 0.4, and 0.5 years of age, respectively. Patients No. I-2, I-6, and I-7 also received HSCT at 1.5, 0.9, and 0.6 years of age, respectively. After ERT and/or HSCT, IDS enzyme activity and urinary DS and HS quantities all significantly improved in all of these patients compared with the baseline data (Figure 4). 

The other six patients were regularly followed up at our clinic every 6 months to monitor for any emerging MPS symptoms. At the time of this study, none of these subjects have developed significant symptoms. Table 2 shows the long-term follow-up data compared with the baseline data of the infants in Group 1. We chose one representative infant with the longest follow-up time with each *IDS* variant and reported the findings as follows.

### 3.2. Variation Allele c.254C > T [p.A85V]

One infant (No. I-1) had this variation allele. The ACMG classification of this variant is pathogenic. We previously reported that the COS-7 cell construct expressed low activity of around 22.6% of that of the wild-type [28]. At 0.4 years of age, his IDS enzyme activity was 0.83 µmol/g protein/4 h (reference range 12.89–131.83). Urinary GAG biochemistry examinations revealed positive results. At 3.9 years of age, his IDS enzyme activity was 1.24 µmol/g protein/4 h. Urinary GAG biochemistry examinations were still positive; however, his physical examinations, bone X-ray, echocardiography, and abdominal ultrasonography were all normal.

### 3.3. Variation Allele c.311A > T [p.D104V]

**Figure 5:**
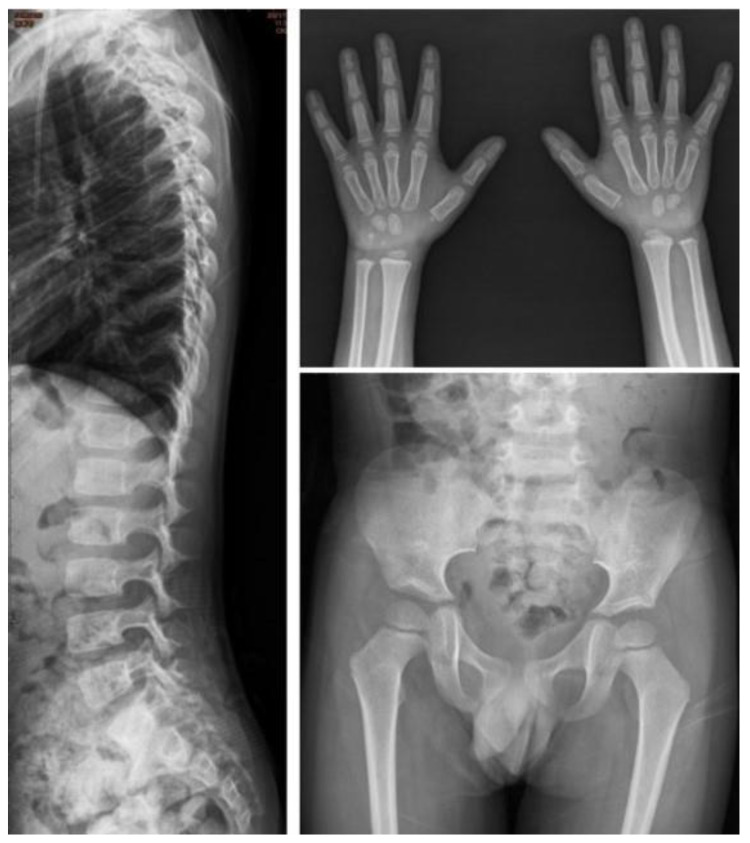
The bone X-rays at 4.3 years of age of patient No. I-2 who had the variation allele c.311A > T [p.D104V]. ERT was started at 1 year of age. He received HSCT at 1.5 years of age. At 4.3 years of age, X-rays of his spine, hands, and pelvis were all normal.

One infant (No. I-2) had this variation allele. The ACMG classification of this variant is pathogenic. We previously reported that the COS-7 cell construct expressed a low activity of around 0% of that of the wild-type [28]. ERT was started at 1 year of age, and he had a good outcome after receiving HSCT at 1.5 years of age. At 2.9 years of age, his peripheral blood *IDS* genotype turned to c.311A (wild-type). He had 95% engraftment and normal blood leukocyte IDS enzyme activity of 124.88 μmol/gm protein/4 h at 4.3 years of age. His urinary DS and HS levels also decreased from 15.62 to 0.01 µg/mL (reference range <0.80), and from 103.44 to 5.89 µg/mL (reference range <0.78), respectively. X-rays of his spine, hands, and pelvis at 4.3 years of age were all normal (Figure 5).

### 3.4. Variation Allele c.817C > T [p.R273W]

Three infants had this variation allele. The ACMG classification of this variant is of uncertain significance. We previously reported that the COS-7 cell construct expressed low activity of around 0% of that of the wild-type [28]. At 0.1 years of age, subject I-3.1 had IDS enzyme activity of 0.2 µmol/g protein/4 h. Urinary GAG biochemistry examinations revealed positive results. At 4.8 years of age, his IDS enzyme activity was 19.69 µmol/g protein/4 h. Urinary GAG biochemistry examinations showed negative results except for a high HS level (41.56 µg/mL). Subject I-3.2, his elder brother, also had this variation allele. At 8.7 years of age, his IDS enzyme activity was 21.5 µmol/g protein/4 h. Urinary GAG biochemistry examinations showed negative results except for a high HS level (17.72 µg/mL). However, their physical examinations, bone X-ray, echocardiography, and abdominal ultrasonography were all normal.

### 3.5. Variation Allele c.1025A > G [p.H342R]

Two infants had this variation allele. The ACMG classification of this variant is pathogenic. We previously reported that the COS-7 cell construct expressed low activity of around 41.8% of that of the wild-type [28]. At 0.1 years of age, subject I-4.1 had IDS enzyme activity of 0.4 µmol/g protein/4 h. Urinary GAG biochemistry examinations revealed positive results. At 3.6 years of age, his IDS enzyme activity was 11.2 µmol/g protein/4 h. Urinary GAG biochemistry examinations showed negative results except for a high HS level (17.72 µg/mL). Subject I-4.2, his elder brother, also had this variation allele. At 6.1 years of age, his IDS enzyme activity was 9.05 µmol/g protein/4 h. Urinary GAG biochemistry examinations showed negative results except for a high HS level (16.72 µg/mL). Their physical examinations, bone X-ray, echocardiography, and abdominal ultrasonography were all normal except for dysrhythmia in the older brother.

### 3.6. Variation Allele c.1400C > T [p.P467L]

**Figure 6:**
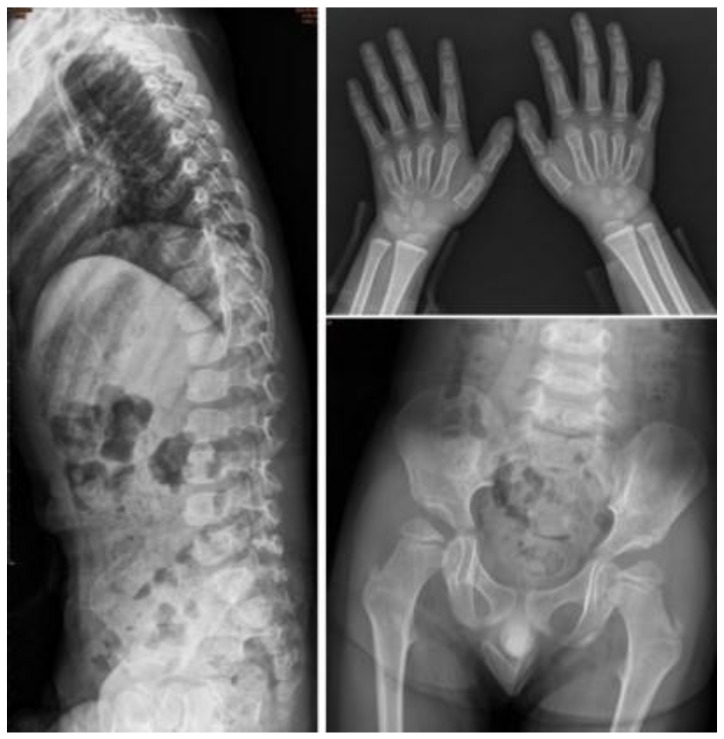
The bone X-rays at 3.2 years of age of patient No. I-5 who had the variation allele c.1400C > T [p.P467L]. He received ERT at 0.5 years of age. At 3.2 years of age, rounded and anterior beaking of lumbar spine vertebral bodies, mild proximal tapering of metacarpal bones with bullet-shaped phalanges, round iliac wings, and inferior tapering of the ilea with not-well-developed acetabulum were found.

One infant (No. I-5) had this variation allele. The ACMG classification of this variant is pathogenic. We previously reported that the COS-7 cell construct expressed low activity of around 0% of that of the wild-type [28]. His two maternal uncles had also been MPS II patients and died when his mother was in elementary school. He received ERT at 0.5 years of age, after which the urinary GAGs significantly improved. A bone X-ray at 3.2 years of age revealed rounded and anterior beaking of lumbar spine vertebral bodies, mild proximal tapering of metacarpal bones with bullet-shaped phalanges, round iliac wings, and inferior tapering of the ilea with not-well-developed acetabulum (Figure 6).

### 3.7. Variation Allele c.1007–1666_c.1180 + 2113 delinsTT

**Figure 7:**
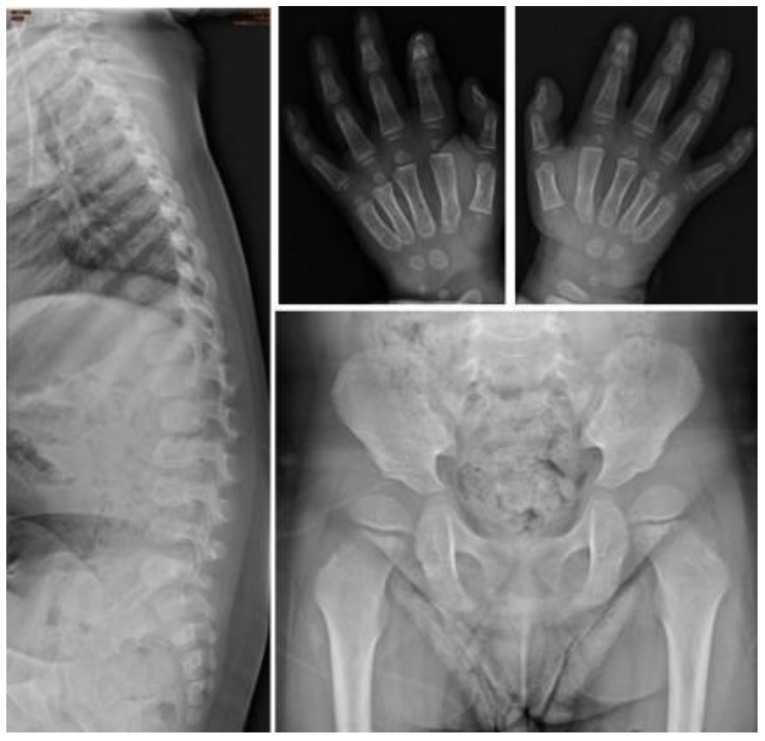
The bone X-rays at 2.8 years of age of patient No. I-6 who had the variation allele c.1007–1666_c.1180 + 2113 delinsTT. ERT was started at 0.4 years of age. He received HSCT at 0.9 years old. At 2.8 years of age, anterior beaking of lower thoracic to lumbar vertebrae, proximal pointed metacarpal and bullet-shaped phalanges of both hands, and shallowed bilateral acetabuli and coxa valga of both femurs were found.

One infant (No. I-6) had this variation allele. The ACMG classification of this variant is pathogenic. ERT was started at 0.4 years of age, and he received HSCT at 0.9 years of age. He developed some complications after HSCT with manifestations of severe edema, partial graft-versus-host disease (GVHD), and liver function impairment with jaundice; however, his condition was managed properly thereafter. At 1.2 years of age, his peripheral blood *IDS* genotype turned to the wild-type. At 2.8 years of age, he had normal blood leukocyte IDS enzyme activity of 110.92 μmol/gm protein/4 h. His urinary GAG biochemistry examinations also significantly improved compared with the baseline data at 0.1 years of age. A bone X-ray at 2.8 years of age showed anterior beaking of lower thoracic to lumbar vertebrae, proximal pointed metacarpal and bullet-shaped phalanges of both hands, and shallow bilateral acetabuli and coxa valga of both femurs (Figure 7).

### 3.8. Variation Allele IDS Inversion

One infant (No. I-7) had this variation allele. ERT was started at 0.5 years of age after which the urine GAG quantities significantly improved. He received HSCT at 0.6 years of age; however, he died at 0.8 years of age due to infection and sepsis.

### 3.9. Group 2: Suspected MPS II or Pseudodeficiency

Nine *IDS* gene variants were identified in this group. A total of 151 infants in this group were “negative” for urinary GAG biochemistry examinations, “deficient” for IDS enzymatic activity, and had at least one hemizygous variant of the *IDS* gene.

### 3.10. Variation Allele c.589C > T [p.P197S]

One infant (No. II-1) had this variation allele (Table 3). The ACMG classification of this variant is likely pathogenic. We previously reported that the COS-7 cell construct expressed high activity of around 74.9% of that of the wild-type [28]. At 0.2 years of age, his IDS enzyme activity was 7.80 µmol/g protein/4 h. Urinary GAG levels were normal except for a small elevation in HS level (1.46 µg/mL) (reference range <0.78). At 5.6 years of age, his IDS enzyme activity was 25.08 µmol/g protein/4 h. There was still a small elevation in HS level (1.82 µg/mL). His physical examinations, bone X-ray, echocardiography, and abdominal ultrasonography were all normal.

### 3.11. Variation Allele c.659T > C [p.F220S]

Two infants had this variation allele. The ACMG classification of this variant is likely pathogenic. The COS-7 cell construct expressed low activity of around 0% of that of the wild-type in the present study. With regards to maternal-side male senior relative screening, a 56-year-old maternal grandfather of one suspected infant (No. II-2) was also identified to have this variant. His physical examinations, IDS enzyme activity, and urinary GAG quantities were all normal except for a small elevation in HS level (0.88 µg/mL) (reference range < 0.78) (Table 3 and Table 4).

### 3.12. Variation Allele c.778C > T [p.P260S]

One infant (No. II-3) had this variation allele. The ACMG classification of this variant is likely pathogenic. We previously reported that the COS-7 cell construct expressed a high activity of around 84.5% of that of the wild-type [28]. At 0.1 years of age, his IDS enzyme activity was 6.47 µmol/g protein/4 h. Urinary GAG levels were normal. At 3 years of age, his IDS enzyme activity was 9.27 µmol/g protein/4 h. Urinary GAGs, physical examinations, bone X-ray, echocardiography, and abdominal ultrasonography were all normal.

### 3.13. Variation Allele c.851C > T [p.P284L]

Four infants had this variation allele. The ACMG classification of this variant is of uncertain significance. We previously reported that the COS-7 cell construct expressed high activity of around 62.3% of that of the wild-type [28]. Infant No. II-4 had the longest follow-up period among these four infants. At 0.1 years of age, his IDS enzyme activity was 0.51 µmol/g protein/4 h. Urinary GAG quantities were normal. At 3.4 years of age, his IDS enzyme activity was 20.79 µmol/g protein/4 h. Urinary GAGs, physical examinations, bone X-ray, echocardiography, and abdominal ultrasonography were all normal. This variation allele has been reported to be an attenuated phenotype [37]; however, Sawada et al. reported at the ACIMD conference in 2012 that this variation allele might cause structural modeling of the IDS enzyme and result in pseudodeficiency [24].

### 3.14. Variation Allele c.890G > A [p.R297H]

Two infants had this variation allele. The ACMG classification of this variant is pathogenic. We previously reported that the COS-7 cell construct expressed high activity of around 98.9% of that of the wild-type [28]. Since these two infants were both younger than 6 months old at the time of this study, longer-term follow-up examinations are warranted.

### 3.15. Variation Allele c.1513T > C [p.F505L]

One infant (No. II-6) had this variation allele. The ACMG classification of this variant is likely pathogenic. The COS-7 cell construct expressed high activity of around 84.6% of that of the wild-type in the present study. At 0.1 years of age, his IDS enzyme activity was 5.93 µmol/g protein/4 h. Urinary GAG levels were normal. At 3 years of age, his IDS enzyme activity was 15.82 µmol/g protein/4 h. Urinary GAGs, physical examinations, bone X-ray, echocardiography, and abdominal ultrasonography were all normal.

### 3.16. Variation Alleles c.851C > T [p.P284L]; c.1180 + 184T > C

One infant (No. II-7) had the combination of these two variation alleles. Since he was younger than 6 months old at the time of this study, longer-term follow-up examinations are warranted.

### 3.17. Variation Alleles c.103 + 34_56dup; c.684A > G [p.Pro228 =]; c.851C > T [p.P284L]; c.1180 + 184T > C

**Figure 8:**
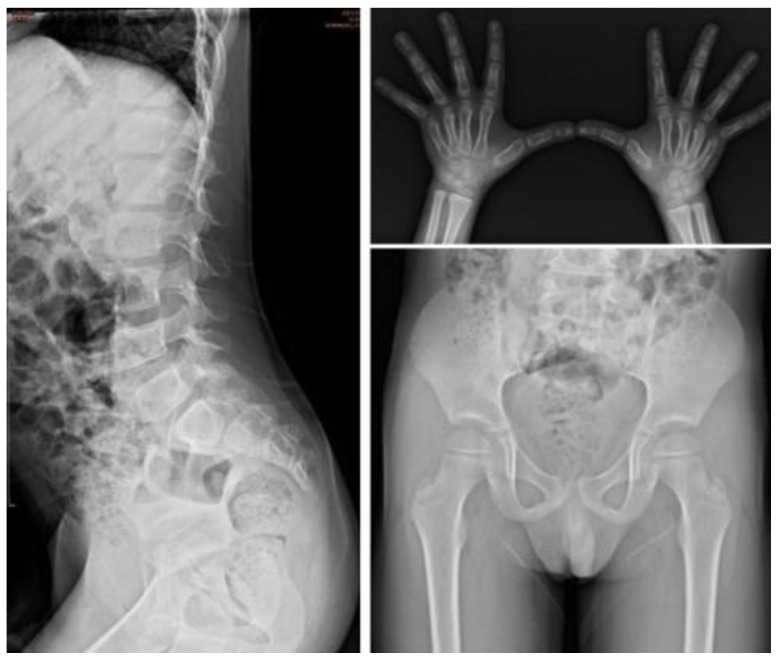
The bone X-rays at 6.1 years of age of subject No. II-8 who had the variation alleles c.103 + 34_56dup; c.684A > G [p.Pro228=]; c.851C > T [p.P284L]; c.1180 + 184T > C. At 6.1 years of age, X-rays of his spine, hands, and pelvis were all normal.

One hundred and thirty-nine infants (68.8%) had the combination of these four variation alleles. The IDS enzyme activity was 6.1 ± 5.2 (mean ± standard deviation) µmol/g protein/4 h. Urinary GAG examinations, including DMB/Cre ratio, DS, HS, and KS levels were all normal. Subject II-8 had the longest follow-up period among these 139 infants. At 0.2 years of age, his IDS enzyme activity was 4.20 µmol/g protein/4 h. Urinary GAG quantities were normal. At 6.1 years of age, his IDS enzyme activity was 13.51 µmol/g protein/4 h. Urinary GAGs, physical examinations, bone X-ray, echocardiography, and abdominal ultrasonography were all normal (Figure 8). 

With regards to maternal-side male senior relative screening, a 90-year-old maternal great grandfather of the suspected infant also had a combination of these four variants. His physical examinations, IDS enzyme activity, and urinary GAG quantities were all normal (Table 4).

### 3.18. Group 3: Non-MPS II

Five *IDS* gene variants were identified in this group. Forty-one infants in this group were “negative” for urinary GAG biochemistry examinations, “normal” for IDS enzymatic activity, and had at least one hemizygous variant of the *IDS* gene (Table 1).

### 3.19. Variation Allele c.142C > T [p.R48C]

Two infants had this variation allele. The ACMG classification of this variant is likely pathogenic. We previously reported that the COS-7 cell construct expressed a high activity of around 83.6% of that of the wild-type [28].

### 3.20. Variation Allele c.301C > T [p.R101C]

Six infants had this variation allele. The ACMG classification of this variant is of uncertain significance. We previously reported that the COS-7 cell construct expressed high activity of around 97% of that of the wild-type [38,39].

### 3.21. Variation Allele c.805G > A [p.D269N]

One infant had this variation allele. However, the ACMG classification of this variant is pathogenic. The COS-7 cell construct expressed low activity of around 0% of that of the wild-type in the present study.

### 3.22. Variation Allele c.1478G > A [p.R493H]

Ten infants (5%) had this variation allele. The ACMG classification of this variant is likely pathogenic. We previously reported that the COS-7 cell construct expressed high activity of around 86.5% of that of the wild-type [28]. With regards to maternal-side male senior relative screening, a 63-year-old maternal grandfather of the suspected infant also had this variant. His physical examinations, IDS enzyme activity, and urinary GAG levels were all normal (Table 4).

### 3.23. Variation Allele c.1499C > T [p.T500I]

Twenty-two infants (11%) had this variation allele. The ACMG classification of this variant is likely benign. We previously reported that the COS-7 cell construct expressed high activity of around 77.5% of that of the wild-type [28].

## 4. Discussion

To the best of our knowledge, this is the first report of timely ERT and/or HSCT for infants diagnosed with MPS II through a newborn screening program with up to 6 years of follow-up data. Four patients received intravenous ERT at 1, 0.5, 0.4, and 0.5 years of age, respectively. Three patients also received HSCT at 1.5, 0.9, and 0.6 years of age, respectively. After ERT and/or HSCT, IDS enzyme activity, urinary DMB/Cre ratio, and DS and HS quantities in these patients all significantly improved compared with their baseline data. Since the commencement of the newborn screening program for MPS II in Taiwan, the issue of the relationship between genotype and phenotype of MPS II has become increasingly important. In this cohort of 202 referred cases, 10 infants with seven *IDS* gene variants were diagnosed with confirmed MPS II (Group 1), 151 infants with nine *IDS* gene variants were classified as having suspected MPS II or pseudodeficiency (Group 2), and 41 infants with five *IDS* gene variants were classified as not having MPS II (Group 3) according to the results of urinary GAG biochemistry examinations, IDS enzymatic activity, and the identification of one hemizygous variant of the *IDS* gene. Long-term follow-up every 6 months was suggested and arranged for the infants in Group 1 and Group 2. 

In Taiwan, the National Health Insurance program was established in 1995. Subsequently, the Rare Diseases and Orphan Drugs Act was approved in 2000, and the national MPS II newborn screening program was launched in 2015 with a median diagnostic age as early as 0.2 years. Therefore, the timely referral of MPS patients to medical specialists and the early application of multidisciplinary care, as well as promising advancements in ERT and HSCT have led to better treatment outcomes.

HSCT is effective in improving some manifestations of MPS II, including endurance, joint mobility, hepatosplenomegaly, upper airway obstruction, respiratory function, and growth [12,40,41,42]. However, this therapy has a restricted effect on existing skeletal dysplasia, cardiac valvular dysplasia, and cognitive impairment. The advantages of HSCT include the ability to cross the blood–brain barrier, that it can improve some skeletal manifestations, permanent treatment and the continuous secretion of enzymes, and the elimination of preexisting immune responses against infused enzymes by ERT. The disadvantages of HSCT include rejection, difficulty in finding matched donors, GVHD, the need for a special facility, and the risk of mortality [16]. Of the three patients who received HSCT in the current study, one had a smooth treatment course, another had some complications and GVHD, and the other died 2 months after HSCT due to infection and sepsis.

ERT for MPS II was approved in 2006 and has demonstrated efficacy in GAG degradation and improvement in clinical symptoms, mainly in patient endurance and visceral organs [43]. The advantages of ERT include low risk, ease of administration, that it does not compromise health, and there is no age limitation. The disadvantages of ERT include the high cost, short enzyme half-life, the need for continuous administration (typically weekly or bi-weekly), that it does not penetrate the blood–brain barrier, and that it is less effective in heart valves, bone, and cornea [16]. Providing ERT before and for weeks after HSCT is required to moderate the common antibody response after transplantation, and to achieve better therapeutic efficacy [44]. In the three patients who received HSCT in this study, a combination of treatments was also used, first with ERT and then with HSCT.

The activities determined in COS-7 constructs correlated with the activities determined in the leukocytes in most subjects in our cohort except for specific variants. It is interesting about the condition of c.805G > A [p.D269N] variant in group 3 which was considered as pathogenic according to ACMG classification; however, the enzyme activity in COS-7 transfected cells was null, while it was normal in leucocytes. Because this is the only one subject with this variant in our cohort as well as the fact that no MPS II patient was reported with this variant in the literature, long-term and comprehensive follow-up for this subject is warranted.

In our study, the reference range of IDS enzyme activity is 12.89~131.83 µmol/g protein/4 h. For patients No. I-2 and I-6, the IDS enzyme activities were all within the reference range after receiving ERT and HSCT. Some decreases of IDS enzyme activity at 2.3, 2.7, and 4.0 years in Figure 4A may be based on the patient’s personal condition at that time (e.g., nutritional status, exercise, and sleep quality).

In Table 3, we observed the situation that the IDS enzyme activities increase in some patients and decrease in others when comparing baseline, and follow-up data may depend on each patient’s personal condition at that time (e.g., age and nutritional status). We focused on if IDS enzyme activity was within the reference range (12.89~131.83 µmol/g protein/4 h).

In MPS disorders, GAGs accumulate in the lysosomes and extracellular matrices of a variety of tissues, and their levels can be measured using different qualitative and quantitative methods in various biological samples, including urine and blood, to investigate the diagnosis, pathogenesis, and prognosis of the disease [45]. The assessment of GAG fractionation biomarkers using the LC-MS/MS method is a reliable and accurate method to simultaneously quantify urinary quantities of DS, HS, and KS, and these biomarkers are more sensitive than the traditional DMB/Cre ratio using the spectrophotometric method to diagnose MPS, identify subgroups, screen high-risk populations, and assess the efficacy of ERT and HSCT [46]. In our cohort, the quantities of three urinary GAGs (DS, HS, and KS) were evaluated using the LC-MS/MS method, and the non-specific total level of urinary GAGs (DMB/Cre ratio) was assessed using the DMB spectrophotometric method to help make a correct diagnosis of MPS II, as well as for long-term follow-up of the suspected subjects for observation, and the effects of ERT and HSCT for the patients who received treatment.

Lin et al. [6] reported that in 61 male Taiwanese patients with MPS II from the Hunter Outcome Survey, the median ages at symptom onset and diagnosis were 2.5 years and 3.5 years, respectively. Hernia, facial features consistent with MPS II, and claw hands were the earliest emerging symptoms, with median ages of 3.2 years, 4.3 years, and 4.7 years, respectively. The median age at first surgery for hernia repair was 4.2 years (*n* = 35). In our cohort, we closely monitored the suspected subjects every 6 months for any emerging symptoms.

Because MPS II in an X-linked recessive disorder, the risk of recurrence is 50% for affected sons and 50% for carrier daughters. It is necessary to investigate whether the occurrence of MPS II in the affected son is inherited from a carrier mother, or whether it is caused by a de novo mutation. Asian countries have a higher incidence of MPS II among the various types of MPS disorders. Because almost all MPS II patients are males and all carriers are females, the diagnosis of MPS II can be disturbing to families due to the patriarchal society in Asia. Thus genetic counseling for family members should be conducted in a sensitive manner, and genetic screening needs to be offered to potential female carriers [47]. Among the 21 *IDS* gene variants identified in this study, up to 16 (76%) were novel variants and were only identified in our newborn screening program for MPS II. Therefore, maternal-side male senior relative screening for these novel variants was of particular importance for genetic counseling. In our cohort, three maternal-side male senior relatives were identified as having the same *IDS* variant. Their physical examinations, IDS enzyme activity, and urinary GAG quantities were checked, and the results were all normal. As a result, the natural courses of the subjects with these three *IDS* gene variants are probably benign.

Since MPS II is a rare, progressive, multisystemic disease with insidious initial symptoms, making an early diagnosis can be a great challenge for first-line health care practitioners [28]. Newborn screening programs allow for an early diagnosis for MPS II at an asymptomatic age, and thus for more timely and appropriate management for these patients. In the present study, four patients identified through the MPS II newborn screening program received ERT following their diagnosis, and this treatment was reimbursed by the National Health Insurance program in keeping with international standards. Three of our MPS II patients also received HSCT. Following a thorough explanation of the progressive nature of MPS II and the significance of the early introduction of ERT or HSCT before the occurrence of irreversible organ damage, the parents of the other six subjects determined that their children should receive regular follow-ups every 6 months at our clinic to monitor any emerging manifestations of MPS II. ERT and HSCT can improve or lessen the natural progression of the disease, and early initiation of these treatments may lead to better outcomes [16]. Longer follow-up periods are warranted to observe the efficacy of these treatments in children with MPS II who are diagnosed early through newborn screen programs.

### Limitations

In the current study, it was not easy to categorize the children under five years of age diagnosed through the newborn screening program into MPS II subgroups (severe vs. mild) due to the difficulty in determining their cognitive development status. Consequently, studies with a larger cohort and a longer follow-up period are warranted to observe the clinical outcomes of these children.

## 5. Conclusions

Because of the progressive nature of MPS II, an early diagnosis via a newborn screening program and timely initiation of ERT and/or HSCT before the occurrence of irreversible organ damage may lead to better clinical outcomes. The findings of this study could serve as baseline data for the analysis of any emerging symptoms over a longer follow-up period for suspected MPS II subjects, as well as the long-term effects of ERT and HSCT in the patients with MPS II, and could help develop quality of care strategies.

## Figures and Tables

**Table 1:** Variation alleles of the *IDS* gene, IDS enzyme activity, biochemistry GAG tests, and management of the 202 referred subjects from our MPS II newborn screening program categorized into three diagnostic groups as follows: Group 1 (confirmed MPS II), Group 2 (suspected MPS II or pseudodeficiency), and Group 3 (non-MPS II).

Diagnostic Group	Variation Allele of *IDS* Gene	Known/Novel	ACMG Classification	IDS Activity Expressed in Transfected COS-7 Cells (%)	*N*	%	Gender	IDS Enzyme Activity (µmol/g Protein/4 h)	Urinary DMB/Cre Ratio (mg/mmol Creatinine)	DS (µg/mL)	HS (µg/mL)	KS (µg/mL)	Management
Group 1	c.254C > T, p.A85V	Novel	Pathogenic	22.6%	1	0.5%	M	0.83	78.58	11.59	12.36	0.25	Regular follow-up
	c.311A > T, p.D104V	Novel	Pathogenic	2.2%	1	0.5%	M	0.32	113.95	15.62	103.44	1.42	ERT + HSCT
	c.817C > T, p.R273W	Novel	Uncertain Significance	2.2%	3	1.5%	M	3.25 ± 2.64	69.71 ± 9.44	3.18 ± 3.65	20.0 ± 19.37	2.92 ± 2.92	Regular follow-up
	c.1025A > G, p.H342R	Novel	Pathogenic	41.8%	2	1.0%	M	1.26/0.40	59.46/70.9	1.18/21.21	8.22/12.06	1.49/6.47	Regular follow-up
	c.1400C > T, p.P467L	Known [32,33]	Pathogenic	0	1	0.5%	M	0.27	153.16	21.4	30.01	0.11	ERT
	c.1007–1666_c.1180 + 2113 delinsTT	Known [34,35]	Pathogenic		1	0.5%	M	0.99	177.96	30.77	203.35	0.31	ERT + HSCT
	IDS inversion	Known [18,36]			1	0.5%	M	0.13	104.84	11.93	175.36	0.15	ERT + HSCT
Group 2	c.589C > T, p.P197S	Novel	Likely Pathogenic	74.9%	1	0.5%	M	7.8	63.82	0.38	1.46	6.35	Regular follow-up
	c.659T > C, p.F220S	Novel	Likely Pathogenic	0	2	1.0%	M	10.36/7.99	73.2/89.81	0.21/0.19	3.69/5.89	4.03/5.05	Regular follow-up
	c.778C > T, p.P260S	Novel	Likely Pathogenic	84.5%	1	0.5%	M	6.47	12.29	0.12	0.1	1.24	Regular follow-up
	c.851C > T, p.P284L	Known	Uncertain Significance	62.3%	4	2.0%	M	8.7 ± 8	27.9 ± 5.5	0.03	0.12 ± 0.05	0.22 ± 0.02	Regular follow-up
	c.890G > A, p.R297H	Novel	Pathogenic	98.9%	2	1.0%	M	9.2/58.75	69.67/50.38	0.08/0.03	0.04/0.6	3.47/2.5	Regular follow-up
	c.1513T > C, p.F505L	Novel	Likely Pathogenic	84.6%	1	0.5%	M	5.93	27.69	0.18	0.2	0.08	Regular follow-up
	c.851C > T, p.P284L; c.1180 + 184T > C	Known [37]; Novel	Uncertain Significance + (–)		1	0.5%	M	2.93	55.87	0.14	0.38	0.32	Regular follow-up
	c.103 + 34_56dup; c.684A > G, p.Pro228 =; c.851C > T, p.P284L; c.1180 + 184T > C	Novel; Novel; Known [37]; Novel	Uncertain Significance + Likely Benign + Uncertain Significance + (–)		139	68.8%	M	6.1 ± 5.2	27.0 ± 7.8	0.16 ± 0.18	0.29 ± 0.22	1.22 ± 1.74	Regular follow-up
Group 3	c.142C > T, p.R48C	Novel	Likely Pathogenic	83.6%	2	1.0%	M	16.27/23.78	38.73/26.2	0.01/0.08	0.75/0.38	0.03/0.59	Observation
	c.301C > T, p.R101C	Known [38]	Uncertain Significance	97%	6	3.0%	M	26.1 ± 9.9	18.1 ± 6.4	0.06 ± 0.04	0.14 ± 0.04	4.92 ± 1.9	Observation
	c.805G > A, p.D269N	Novel	Pathogenic	0	1	0.5%	M	17.68	46.09	0.17	0.53	1.32	Observation
	c.1478G > A, p.R493H	Novel	Likely Pathogenic	86.5%	10	5.0%	M	35.2 ± 34.9	34.6 ± 15.4	0.16 ± 0.16	0.18 ± 0.21	1.60 ± 2.78	Observation
	c.1499C > T, p.T500I	Novel	Likely Benign	77.5%	22	10.9%	M	25 ± 10.8	30.3 ± 10.8	0.09 ± 0.06	0.1 ± 0.09	0.36 ± 0.26	Observation

IDS enzyme activity (reference range 12.89~131.83 µmol/g protein/4 h); GAG quantification (DMB/Cre ratio) and the normal reference values based on the age groups: <6 months: <70.68 mg/mmol creatinine; 6 months–2 years: <46.80 mg/mmol creatinine; 2–17 years: <20.98 mg/mmol creatinine; and >18 years: <12.62 mg/mmol creatinine. Quantitative analyses of GAG-derived disaccharides (DS, HS, or KS) by tandem mass spectrometry assay. The normal cut-off values: DS < 0.80 µg/mL; HS < 0.78 µg/mL; and KS < 7.90 µg/mL. IDS, iduronate-2-sulfatase; DS, dermatan sulfate; HS, heparan sulfate; KS, keratan sulfate; ERT, enzyme replacement therapy; HSCT, hematopoietic stem cell transplantation.

**Table 2:** The baseline and long-term follow-up data of Group 1 (confirmed MPS II). We chose one subject with the longest follow-up time with each *IDS* gene variant as the representative example.

No.	Gender	Variation Allele of *IDS* Gene	Age at HSCT (Years)	Age at Start of ERT (Years)	Baseline/Follow-Up	Age (Years)	IDS Enzyme Activity (µmol/g Protein/4 h)	Urinary DMB/Cre Ratio (mg/mmol Creatinine)	DS (µg/mL)	HS (µg/mL)	KS (µg/mL)	Hand X-ray	L-S Spine X-ray	Pelvis X-ray	Echocardiography	Abdominal ultrasonography
I-1	M	c.254C > T, p.A85V	—	—	Baseline	0.4	0.83	78.58	11.59	12.36	0.25	Normal	Normal	Normal	Normal	Normal
					Follow-up	3.9	1.24	35.42	0.77	25.75	4.46	Normal	Normal	Normal	Normal	Normal
I-2	M	c.311A > T, p.D104V	1.5	1.0	Baseline	0.1	0.32	113.95	15.62	103.44	1.42	Normal	Normal	Normal	Normal	Normal
					Follow-up	4.3	124.88	13.87	0.01	5.89	0.16	Normal	Normal	Normal	Normal	Normal
I-3.1	M	c.817C > T, p.R273W	—	—	Baseline	0.1	0.2	77.3	7.39	1.83	6.13	Normal	Normal	Normal	Normal	Normal
					Follow-up	4.8	19.69	18.86	0.16	41.56	4.71	Normal	Normal	Normal	Normal	Normal
I-3.2	M	c.817C > T, p.R273W	—	—	Baseline	4.2	0.41	16.73	5.48	24.9	0.58	Normal	Normal	Normal	Normal	Normal
					Follow-up	8.7	21.5	15.64	0.13	17.2	2.29	Normal	Normal	Normal	Normal	Normal
I-4.1	M	c.1025A > G; p.H342R	—	—	Baseline	0.1	0.40	70.9	21.21	12.06	6.47	Normal	Normal	Normal	Normal	Normal
					Follow-up	3.6	11.2	21.69	0.64	17.72	0.49	Normal	Normal	Normal	Normal	Normal
I-4.2	M	c.1025A > G; p.H342R	—	—	Baseline	2.8	NA	108.18	3.81	10.21	4.20	Normal	Normal	Normal	Dysrhythmia	Normal
					Follow-up	6.1	9.05	17.54	0.22	16.72	0.57	Normal	Normal	Normal	Dysrhythmia	Normal
I-5	M	c.1400C > T, p.P467L	—	0.5	Baseline	0.2	0.27	153.16	21.4	30.01	0.11	Proximal tapering of metacarpal bone with bullet-shaped phalanges.	Normal	Normal	Normal	Normal
					Follow-up	3.5	7.51	18.01	0.42	3.28	2.39	Suspicious of mild proximal tapering of metacarpal bone with bullet-shaped phalanges.	Multiplex dysostosis of the spine. L-spine vertebral bodies are round. The anterior beaking more at several vertebral bodies of L-spine.	Round iliac wings, inferior tapering of the ilea with not-well developed acetabulum.	Normal	Normal
I-6	M	c.1007–1666_c.1180 + 2113 delinsTT	0.9	0.4	Baseline	0.1	0.99	177.96	30.77	203.35	0.31	Normal	Normal	Normal	ASD II	Normal
					Follow-up	2.8	110.92	8.9	0.1	20.64	7.05	Persistent proximal pointed metacarpal and bullet-shaped phalanges of both hands.	Anterior beaking of lower thoracic to lumbar vertebrae. Relative enlargement of sternal end of bilateral clavicles.	Shallow bilateral acetabuli and coxa valga of both femurs.	ASD II, MR, AR	Mild splenomegaly
I-7	M	IDS inversion	0.6	0.5	Baseline	0.1	0.13	104.84	11.93	175.36	0.15	NA	NA	NA	NA	NA
					Follow-up	0.6	1.93	44.05	8.72	37.3	0.2	NA	NA	NA	NA	NA

IDS enzyme activity (reference range 12.89~131.83 µmol/g protein/4 h); GAG quantification (DMB/Cre ratio) and the normal reference values based on the age groups: <6 months: <70.68 mg/mmol creatinine; 6 months–2 years: <46.80 mg/mmol creatinine; 2–17 years: <20.98 mg/mmol creatinine; and >18 years: <12.62 mg/mmol creatinine. Quantitative analyses of GAG-derived disaccharides (DS, HS, or KS) by tandem mass spectrometry assay. The normal cut-off values: DS < 0.80 µg/mL; HS < 0.78 µg/mL; and KS < 7.90 µg/mL. HSCT, hematopoietic stem cell transplantation; ERT, enzyme replacement therapy; IDS, iduronate-2-sulfatase; DS, dermatan sulfate; HS, heparan sulfate; KS, keratan sulfate; NA, not available; ASD, atrial septal defect; MR, mitral regurgitation; AR, aortic regurgitation.

**Table 3:** The baseline and long-term follow-up data of Group 2 (suspected MPS II or pseudodeficiency). We chose one subject of the longest follow-up time with each *IDS* gene variant as the representative example.

No.	Gender	Variation Allele of *IDS* Gene	Baseline/Follow-Up	Age (Years)	IDS Enzyme Activity (µmol/g Protein/4 h)	Urinary DMB/Cre Ratio (mg/mmol Creatinine)	DS (µg/mL)	HS (µg/mL)	KS (µg/mL)
II-1	M	c.589C > T, p.P197S	Baseline	0.2	7.80	63.82	0.38	1.46	6.35
			Follow-up	5.6	25.08	11.57	0.03	1.82	1.07
II-2	M	c.659T > C, p.F220S	Baseline	0.1	10.36	70.32	0.21	3.69	4.03
			Follow-up	0.3	4.57	49.65	0.16	2.02	0.37
II-3	M	c.778C > T, p.P260S	Baseline	0.1	6.47	12.29	0.12	0.1	1.24
			Follow-up	3.0	9.27	8.47	0.04	0.04	5.39
II-4	M	c.851C > T, p.P284L	Baseline	0.1	0.51	34.22	0.03	0.08	0.21
			Follow-up	3.4	20.79	12.13	0.04	0.02	2.63
II-5	M	c.890G > A, p.R297H	Baseline	0.2	58.75	50.38	0.08	0.04	3.47
			Follow-up	—	—	—	—	—	—
II-6	M	c.1513T > C, p.F505L	Baseline	0.1	5.93	32.15	0.08	0.11	7.21
			Follow-up	3.0	15.82	9.38	0.02	0.26	5.61
II-7	M	c.851C > T, p.P284L; c.1180 + 184T > C	Baseline	0.1	2.93	55.87	0.14	0.38	0.32
			Follow-up	—	—	—	—	—	—
II-8	M	c.103 + 34_56dup; c.684A > G, p.Pro228 =; c.851C > T, p.P284L; c.1180 + 184T > C	Baseline	0.2	4.20	41.70	0.30	0.02	2.41
			Follow-up	6.1	13.51	11.68	0.01	0.63	1.37

IDS enzyme activity (reference range 12.89~131.83 µmol/g protein/4 h); GAG quantification (DMB/Cre ratio) and the normal reference values based on the age groups: <6 months: <70.68 mg/mmol creatinine; 6 months–2 years: <46.80 mg/mmol creatinine; 2–17 years: <20.98 mg/mmol creatinine; and >18 years: <12.62 mg/mmol creatinine Quantitative analyses of GAG-derived disaccharides (DS, HS, or KS) by tandem mass spectrometry assay. The normal cut-off values: DS < 0.80 µg/mL; HS < 0.78 µg/mL; and KS < 7.90 µg/mL. IDS, iduronate-2-sulfatase; DS, dermatan sulfate; HS, heparan sulfate; KS, keratan sulfate.

**Table 4:** Age, IDS enzyme activity, and biochemistry GAG tests of three senior male relatives of the referred infants in our MPS II newborn screening program who were identified to have the same *IDS* variant.

Diagnostic Group	Gender	Age (Years)	Variation Allele of *IDS* Gene	IDS Enzyme Activity (µmol/g Protein/4 h)	Urinary DMB/Cre Ratio (mg/mmol Creatinine)	DS (µg/mL)	HS (µg/mL)	KS (µg/mL)
Group 2	M	90	c.103 + 34_56dup; c.684A > G, p.Pro228=; c.851C > T, p.P284L; c.1180 + 184T > C	2.23	2.82	0.52	0.21	0.35
Group 2	M	56	c.659T > C, p.F220S	7.50	9.21	0.09	0.88	0.16
Group 3	M	63	c.1478G > A, p.R493H	27.99	1.39	0.11	0.40	0.28

IDS enzyme activity (reference range 12.89~131.83 µmol/g protein/4 h); GAG quantification (DMB/Cre ratio) and the normal reference values based on the age groups: <6 months: <70.68 mg/mmol creatinine; 6 months–2 years: <46.80 mg/mmol creatinine; 2–17 years: <20.98 mg/mmol creatinine; and >18 years: <12.62 mg/mmol creatinine. Quantitative analyses of GAG-derived disaccharides (DS, HS, or KS) by tandem mass spectrometry assay. The normal cut-off values: DS < 0.80 µg/mL; HS < 0.78 µg/mL; and KS < 7.90 µg/mL. IDS, iduronate-2-sulfatase; DS, dermatan sulfate; HS, heparan sulfate; KS, keratan sulfate.

## Data Availability

Not applicable.

## Abbreviations

MPS	mucopolysaccharidosis
IDS	iduronate-2-sulfatase
GAGs	glycosaminoglycans
DS	dermatan sulfate
HS	heparan sulfate
HSCT	hematopoietic stem cell transplantation
ERT	enzyme replacement therapy
KS	keratan sulfate
CS	chondroitin sulfate
LC-MS/MS	liquid chromatography/tandem mass spectrometry
GVHD	graft-versus-host disease
